# Durchführung von digitalen Arbeitssituationsanalysen für die mobil-flexible Arbeit zur Erhebung von psychischen Belastungsfolgen: Befragung von Mitarbeiter*innen einer öffentlichen Verwaltung zu Homeoffice – Erfahrungen während der SARS-CoV-2-Pandemie

**DOI:** 10.1007/s40664-022-00460-2

**Published:** 2022-04-25

**Authors:** Ronja Bölsch-Peterka, Martin Krowicki, Aliena Schmidtke, Irina Böckelmann

**Affiliations:** Bereich Arbeitsmedizin, Medizinische Fakultät, Otto-von-Guericke Universität Magdeburg, Leipziger Str. 44, 39120 Magdeburg, Deutschland

**Keywords:** Qualitative Personalbefragung, Psychische Belastung, Zeit- und ortsunabhängiges Arbeiten, Homeoffice, SARS-CoV-2-Pandemie, Öffentliche Verwaltung, Qualitative staff survey, Psychological stress, Work independent of time and place, Home office, SARS-CoV-2-pandemic, Public administration

## Abstract

**Hintergrund:**

Im Zuge der SARS-CoV-2-Pandemie wurde die Arbeit in vielen Betrieben nach Hause verlagert und innerhalb kurzer Zeit hat sich die Arbeitssituation der Beschäftigten schnell verändert. Um mögliche Belastungen zu identifizieren und gesundheitliche Ressourcen auszubauen, sind fundierte Analysen der Arbeitssituation notwendig.

**Ziel der Arbeit:**

Ziel dieser Arbeit war es, auch in Zeiten mit Kontaktbeschränkungen, fundierte Analysen im Bereich des Betrieblichen Gesundheitsmanagements anzubieten. Mithilfe von digitalen Arbeitssituationsanalysen (ASITA) sollte die Homeoffice-Tätigkeit in einer öffentlichen Verwaltung erfasst und Handlungsempfehlungen abgeleitet werden.

**Material und Methoden:**

Mittels digitaler ASITAs wurden 3 Gruppen (16 Beschäftigte) zu ihrer Arbeitssituation im Homeoffice befragt. Eingeschlossen wurden Beschäftigte, die innerhalb der letzten 12 Wochen vor der Befragung an mindestens 2 Tagen pro Woche im Homeoffice gearbeitet haben.

**Ergebnisse:**

Das Arbeiten im Homeoffice stellt Anforderungen an die Beschäftigten, die wiederum positive als auch negative Beanspruchungsfolgen mit sich bringen. Aufseiten der Arbeitsorganisation ergaben sich individuelle Vorteile, wie der Wegfall von Arbeitswegen oder eine bessere Zeiteinteilung. Hinsichtlich der Kommunikation im Unternehmen fehlten klare Strukturen zur Arbeit im Homeoffice.

**Diskussion:**

Digitale ASITAs können ein aussagekräftiges Instrument sein, um gesundheitliche Belastungen und Ressourcen auch in Unternehmen mit mobil-flexiblen Arbeitsmodellen zu untersuchen. Wie am Beispiel der Pandemie gezeigt, bedingt ein Wandel der Arbeitssituation einen kontinuierlichen Reflexionsprozess, bei dem die Gesundheit der Beschäftigten eine wesentliche Säule sein muss.

## Abstract (en)

**Background:**

In the course of the COVID-19 pandemic, work in many companies was relocated to the home and the work situation of the employees changed suddenly and within a short period of time. In order to identify possible stressors and to expand health resources, a multifactorial analysis of the work situation is necessary.

**Objective:**

The goal of this work was to provide an in-depth analysis in the area of occupational health management even in times of contact restrictions. With the help of digital work situation analysis (ASITA) both stressors and strengths of the home office activity in a public administration were identified and recommendations for appropriate action were derived.

**Material and methods:**

Using digital ASITAs, 3 groups (in total 16 employees) were asked about their work situation and conditions at home. Employees who worked at least 2 days per week from home during the last 12 weeks before the survey were included in the studies.

**Results:**

Working from home places new demands on employees, which in turn lead to both positive and negative consequences. An example of a positive change can be seen with respect to the work organization. Employees reported many advantages, such as the reduction of time commuting to work, and better time management. An example of a negative outcome can be seen in terms of communication in the company, where clear structures are lacking for working in the home office.

**Discussion:**

Digital work situation analysis can be an instrumental tool to examine health burdens and benefits, even in companies with mobile flexible work models. As shown by the pandemic, a change in the work situation within companies requires a continuous process of reflection, a process in which the health of the employees must be an essential part.

Mit der Entwicklung von Informations- und Kommunikationstechnologien haben sich immer mehr Formen der orts- und zeitflexiblen Arbeit herausgebildet. Nie zuvor war Arbeit so vielfältig und individuell: Telearbeit, mobile Arbeit, Gleitzeit, Freelancing oder Coworking sind neue Arbeitsmodelle, welche aus der fortschreitenden Technologisierung und Digitalisierung hervorgegangen sind.

Grundsätzlich ist orts- und zeitflexibles Arbeiten durch eine räumliche und/oder zeitliche Entgrenzung charakterisiert. Arbeit kann nun überall dort stattfinden, wo eine digitale Vernetzung gesichert ist, sei es zu Hause, beim Kunden, im Zug, am Flughafen oder im Hotel. Auch klare zeitliche Grenzen zwischen Arbeit und Freizeit verschwimmen. Der Zugang zu Arbeit ist dadurch in den späten Abendstunden, an Wochenenden oder Feiertagen möglich [19, 20].

Diese Arbeitsmodelle bringen Chancen und Risiken für Arbeitnehmer*innen, Arbeitgeber*innen sowie Wissenschaft und Politik mit sich. Arbeitgeber*innen müssen sich mit dieser Vielzahl an Arbeitsmodellen beschäftigen und diese bestmöglich mit der bestehenden Arbeitsorganisation verknüpfen. Es entstehen neue Herausforderungen für Arbeitgeber*innen und den Arbeits- und Gesundheitsschutz, wie z. B. in einer Anpassung der Gefährdungsbeurteilung psychischer Belastungen um Kriterien der mobil-flexiblen Arbeit. Diese Arbeitsform löst sich von festen Strukturen und wird organisatorisch zunehmend den Beschäftigten überlassen. Damit geht eine hohe Eigenverantwortung einher, die einerseits mehr Flexibilität in der Vereinbarkeit von Beruf und Familie bietet, aber andererseits auch ein hohes Maß an Selbstorganisation verlangt. Aus diesem Grund ist es sinnvoll, einen ganzheitlichen Ansatz zur gesundheitsförderlichen Gestaltung der mobil-flexiblen Arbeit zu verfolgen, der auch den Blickwinkel auf das Job-Demands-Resources-Modell behält [4, 8].

Die Wissenschaft muss sich diesem ständigen Wandel der Arbeitswelt anpassen. Es ergeben sich fortlaufend neue Fragestellungen bezüglich der gesundheitlichen Auswirkungen der orts- und zeitflexiblen Arbeit, die auch die Arbeitsmedizin vor neue Herausforderungen stellen werden. Die empirischen Einflussfaktoren erweisen sich als sehr komplex, und eine systematische Erfassung aller Wirkungszusammenhänge durch quantitative Untersuchungen ist nur bedingt möglich [1].

## Homeoffice in der SARS-CoV-2-Pandemie

Die SARS-CoV-2-Pandemie ist ein Beschleuniger für die digitale Entwicklung von Unternehmen. So wurden im Zuge der Pandemie gute Ausgangsbedingungen für die Arbeit im Homeoffice geschaffen, IT-Infrastrukturen erweitert und Formen der digitalen Zusammenarbeit in virtuellen bzw. hybriden Teams etabliert.

Im Besonderen hat sich Homeoffice als eine Maßnahme zur Reduzierung betriebsbedingter Personenkontakte in Zeiten erhöhter Ansteckungsrisiken etabliert und wurde im Rahmen der SARS-CoV-2-Arbeitsschutzverordnung gesetzlich festgehalten [6]. Arbeitgeber*innen wurden verpflichtet, Homeoffice anzubieten, soweit keine betrieblichen Gründe entgegenstanden, um der Ausbreitung der SARS-CoV-2-Pandemie und steigender Infektionszahlen entgegenzuwirken.

In der betrieblichen Praxis war der Übergang zu teilweise 100%iger Homeoffice-Arbeit von zahlreichen Anforderungen für Arbeitgeber*innen und Arbeitnehmer*innen geprägt. Der relativ schnelle, teilweise sprunghafte Umstieg machte eine strukturierte Einführung von Homeoffice für viele Unternehmen schwierig.

Noch immer arbeiten Unternehmen die vergangenen Erfahrungen der SARS-CoV-2-Pandemie auf. Chancen und Risiken von Homeoffice aus dieser Zeit müssen gezielt ausgewertet und für die zukünftige Gestaltung von Arbeit (mit Homeoffice) genutzt werden.

Der größte Einflussfaktor auf die psychische Gesundheit der Beschäftigten liegt in der Sensibilisierung aller Beteiligten (Mitarbeiter*innen, Führungskräfte, Arbeitsschutzbeauftragte), um mit den veränderten Arbeitsbedingungen umgehen zu können. Eine gesundheitsförderliche Arbeitsgestaltung und klare gesetzliche Regelungen für den Arbeitsschutz müssen auch im Homeoffice zentrales Element sein. Aus der Literatur lassen sich verschiedene Einflussfaktoren auf die psychische Gesundheit im Homeoffice ableiten [10]:

- Fähigkeit des Mitarbeiters zur Selbstorganisation (hohe Autonomie),
- veränderte Work-Life-Balance (Verschiebung von Arbeit in das private Umfeld),
- veränderte soziale Beziehungen (Entfernung vom Team),
- neue Ansprüche an die Rolle der Führungskraft (Führung digitaler Teams).

Zu beachten ist, dass die Arbeit im Homeoffice vor allem in der Zeit der SARS-Cov-2-Pandemie anderen Rahmenbedingungen (z. B. technische Ausstattung, Arbeitslast, digitale Zusammenarbeit) unterlag. Neuere Forschungsarbeiten [11, 17, 21] liefern hierfür weiterführende Erkenntnisse. Grundsätzlich bleiben die oben aufgezählten Einflussfaktoren auf die psychische Gesundheit auch in Pandemie-Zeiten bestehen.

Im Fehlzeiten-Report [9] wird darauf verwiesen, dass die Implementierung von Telearbeit (hier Homeoffice) systematisch stattfinden muss, wenn die Erfolgschancen dieses Modells erhöht werden sollen. Die Einführung von Telearbeit soll eng mit dem Gesundheitsmanagement verknüpft sein.

Nachdem die ersten großen Ungewissheiten im Rahmen der SARS-CoV-2-Pandemie überstanden sind, ist es nun Aufgabe für Unternehmen, die Arbeit im Homeoffice auf ein stabiles Fundament zu stellen.

## Methode

Es wurden die Anforderungen und Ressourcen der Arbeit im Homeoffice in Pandemie-Zeiten durch ein passendes Analyseverfahren untersucht und entsprechende Maßnahmen zur Verbesserung der gesundheitlichen Situation abgeleitet. Die Grundlage dafür bildete das AOK Gesundheitsprojekt „Digitale Arbeitssituationsanalyse in der öffentlichen Verwaltung“.

Die öffentliche Verwaltungseinrichtung, die an dem Projekt teilgenommen hat, ist eine Verwaltungsbehörde mit rund 110 Mitarbeiter*innen und zwei Geschäftsstellen an zwei Standorten in Sachsen-Anhalt.

Die Corona-Krise hat auch diese Einrichtung vor neue Herausforderungen gestellt, die jedoch die Umsetzung der politischen Forderungen unterstützte, indem sie ihren Beschäftigten das Arbeiten im Homeoffice ermöglichte. Homeoffice wurde zwar bereits vor der Pandemie von den Mitarbeiter*innen genutzt, jedoch nicht in einem vergleichbaren Umfang. Die Führungskräfte nutzten das Homeoffice gelegentlich, während die Mitarbeiter*innen nur vereinzelt im Homeoffice gearbeitet haben. Dazu wurde im Vorfeld ein „fester Tag“ im Rahmen eines Mitarbeitergespräches vereinbart. Es konnte nicht kurzfristig über eine Arbeit im Homeoffice entschieden werden.

Innerhalb des Gesundheitsprojektes wurde der Bedarf nach einer fundierten Analyse der Homeoffice-Situation deutlich. Ausschlaggebend war, dass die Einführung im Zuge der SARS-CoV-2-Pandemie schnell vonstattenging und eine Reflexion bzw. Anpassung des Prozesses bisher ausblieb.

Es stellt sich die Frage, was bei der Gestaltung und Umsetzung von Homeoffice zu beachten ist und welche Ressourcen und Belastungsfolgen damit verbunden sind. Die Arbeitssituationsanalysen sollen neue Erkenntnisse zur derzeitigen Arbeitssituation, insbesondere im Homeoffice, bringen und erste Empfehlungen und Lösungsstrategien für einen zukünftigen Einsatz von Homeoffice und für eine gesundheitsgerechte und sichere Arbeitsplatzgestaltung geben. Dazu ist es wichtig, in einen gemeinsamen Dialog zu kommen und die eigene Arbeitssituation besser zu verstehen. Für das Projekt wurden 3 Gruppen befragt. Die Teilnehmer*innen aus der Gruppe 1 und 2 arbeiteten in der Mitarbeiterebene und in der 3. Gruppen waren alle Bereichsleiter*innen vertreten. Die Gruppenstärken sollten 12 Personen nicht überschreiten, weswegen für die Mitarbeiterebene 2 Gruppen eingeplant wurden. An den Tagen der Befragung standen jedoch weniger Mitarbeiter*innen zur Verfügung als geplant. Für die Gruppen 1 und 2 wurden nur Mitarbeiter*innen eingeladen, die vor der Befragung die letzten 3 Monate (März bis Mai 2021) im Homeoffice gearbeitet haben. Die durchgeführten Analysen basieren auf den grundsätzlichen Ansätzen der Arbeitssituationsanalysen.

Eine Arbeitssituationsanalyse (ASITA) ist ein Instrument, bei dem die Mitarbeiter*innen, als „Experten in eigener Sache“, unter Anleitung durch externe Moderation ihre Arbeitssituation analysieren und konstruktive Lösungsvorschläge zu deren Verbesserung entwickeln. Hierbei stehen sowohl Ressourcen als auch negative Belastungsfolgen im Fokus der Analyse. Die Teilnehmer*innen des Workshops werden somit aktiv in die Veränderungsprozesse eingebunden und berichten, welche Beanspruchungen und Belastungsfolgen sie im beruflichen Alltag wahrnehmen, inwiefern sie unter- oder überfordert sind, ob sie sich am Arbeitsplatz wohlfühlen und ob es aus ihrer Sicht Potenziale für eine Verbesserung der Arbeitssituation gibt [3, 15]. Meyn und Peter [14] definieren Arbeitssituationen dabei als „gleichermaßen subjektive wie objektive Gegebenheiten der Arbeitshandlungen, die sich in Bedeutung und Funktion (Sinn) über die Themen der Arbeitshandlungen, z. B. die konkret gestellten Arbeitsaufgaben, erschließen lassen“.

Bei der ASITA nach Nieder [15] wird die Befragung der Mitarbeiter*innen mit Projektgruppenarbeit und Gruppendiskussion kombiniert. Im Gegensatz zu einer klassischen Mitarbeiterbefragung beinhaltet die ASITA einen Austausch mit den Befragten über die Hintergründe und Prioritäten ihrer Angaben und Aussagen, wodurch ein Vorteil für die Entwicklung konkreter Maßnahmen entsteht. Die ASITA liefert nicht nur wichtige Ergebnisse und Informationen zu der derzeitigen Arbeitssituation in kürzester Zeit, sondern bietet auch Möglichkeiten zur Verbesserung des Betriebsklimas und der Unternehmenskultur. Dafür müssen die Befragten zu ihrer eigenen Arbeitssituation und Einschätzung Stellung beziehen sowie in der Gruppenarbeit ihre Anregungen komprimieren und priorisieren. Eine aktive Teilnahme an der Gruppendiskussion erhöht die Bereitschaft einer konstruktiven Zusammenarbeit und zur Lösung der aufgedeckten Probleme. Transparenz, Nachvollziehbarkeit und Kommunikation sind dabei von enormer Bedeutung, da sie ein zielgerichtetes, gemeinschaftliches Arbeiten unterstützen [15].

Das durch eine Moderation geleitete Befragungskonzept und Gruppendiskussionsverfahren deckt qualitative Daten zur Veränderung und Verbesserung der Arbeitssituation in den Bereichen Arbeitsumgebung, Arbeitstätigkeit, Arbeitsorganisation, Vorgesetzten‑/Führungsverhalten und Gruppen‑/Betriebsklima auf. Verschiedenste Faktoren spielen in den jeweiligen Bereichen eine Rolle und werden in Tab. 1 veranschaulicht [7, 16]:

**Table 1:** 

Einflussfaktoren
*Arbeitsumgebung*
Hitze, Kälte, Zugluft, Nässe, Rutschgefahr, Schmutz, Staub, Gase, Dämpfe, Lärm, Licht
*Arbeitstätigkeit*
Kenntnisse und Kompetenzen (Erfahrungen, Ausbildung), Über- und Unterforderung (geistige und/oder körperliche Belastungen), Monotonie, Geschicklichkeit, Verantwortung, Entscheidungsspielraum, Unfallgefahr, Kontrolle
*Arbeitsorganisation*
Arbeitsaufteilung (Wer? Wie? Was? Wann? Wo? Womit?), Arbeitsspitzen, Leerläufe, Unterbrechungen, Arbeitszeitregelung, Absprachen und Kommunikation mit anderen Abteilungen, Informationsflüsse
*Vorgesetzten‑/Führungsverhalten*
Anerkennung von Leistung, Wertschätzung, Unterstützung, Kontrolle, Motivation, Führungsstil, Förderung von Teamgeist, gerechte Behandlung, Kritik, Mitbestimmungsrecht, Vermittlung bei Konflikten
*Gruppen‑/Betriebsklima*
Stimmungslage (im Unternehmen, der Abteilung, dem Arbeitsbereich, der Gruppe), Konkurrenz, Rivalitäten, gegenseitige Unterstützung, Vertrauen, persönliche Kontakte, Zusammenarbeit, gemeinsame Aktivitäten

Quelle: eigene Darstellung in Anlehnung an [7, 16]

Der erste Teil der ASITA, in dem die Bereiche der Arbeitssituation von den Teilnehmer*innen aus ihrer subjektiven Sicht bewertet werden, ist eine quantitative Datenerhebung. Der Hauptteil der Analyse ist jedoch eine qualitative Evaluation, was den Vorteil hat, dass die Perspektiven der Beteiligten berücksichtigt werden.

Die für die Durchführung der ASITA benötigten Bausteine werden durch die BGN [2] detailliert beschrieben:

Nach einer anfänglichen Begrüßungsrunde erklären die Moderator*innen den Ablauf und die Ziele der Befragung mit entsprechenden Anleitungen auf einem Flipchart. Die Stärken und Ressourcen werden erfasst und von den Moderator*innen für den weiteren Verlauf auf Karten notiert. Im Anschluss werden die Teilnehmer*innen aufgefordert, ihren generellen Veränderungsbedarf mit Klebepunkten sichtbar zu machen, um den Veränderungsbedarf der ASITA einzuschätzen zu können.

Die Moderation wertet den Veränderungsbedarf aus und erläutert anschließend die 5 Bereiche der Arbeitssituation (Arbeitsumgebung, Arbeitstätigkeit, Arbeitsorganisation, Vorgesetzten‑/Führungsverhalten und Gruppen‑/Betriebsklima). Jede*r Workshopteilnehmer*in markiert die Bereiche, die für ihn/sie von größter Bedeutung sind mit Klebepunkten auf einem Whiteboard oder einer Metaplanwand. Jede ASITA ist einzigartig und bringt aufgrund der verschiedenen Ansichten der Teilnehmer neue Aspekte und neues Material für die Diskussion hervor. Die Analyse wird mit den von den Teilnehmer*innen am wichtigsten bewerteten Bereichen fortgeführt. Allerdings kann die Vertiefung der verschiedenen Bereiche nicht gleichzeitig passieren, sodass der am stärksten gewichtete Veränderungsbedarf zuerst bearbeitet wird. In diesem Abschnitt leiten die Moderator*innen die Gruppendiskussion an und die Teilnehmer*innen können ihre Ansichten und Meinungen zum Ausdruck bringen. Diese werden wiederum für alle sichtbar auf der Metaplanwand dokumentiert. Neben konkreten Problemen werden die Teilnehmer*innen auch bereits nach Lösungen für eine bessere Gestaltung der Arbeitssituation gefragt. Im letzten Teil werden die Ergebnisse noch einmal zusammengefasst und für eine Freigabe an die Führungsebene von den Teilnehmer*innen abgesegnet. Beiträge, die nicht an die Führungsebene weitergeleitet werden sollen, werden entsprechend markiert und nicht in das Protokoll übernommen. Zum Abschluss gibt die Moderation einen Ausblick auf den weiteren Prozess, wie z. B. die Aufbereitung und Weiterleitung der Ergebnisse an die Führungsebene für eine Ableitung und Umsetzung von Verbesserungsmaßnahmen.

Nach der Arbeitssituationsanalyse werden die Ergebnisse in einem Ergebnisbericht zusammengefasst und ein Fotoprotokoll erstellt. Anschließend werden die Ergebnisse in einem Ergebnisworkshop mit der Führungsebene kommuniziert und diskutiert. Sofortmaßnahmen werden beschlossen sowie weitere Maßnahmen in einem Maßnahmenplan dokumentiert. Der Maßnahmenplan beinhaltet zusätzlich zu bearbeitende Themen, deren Termine sowie die Regelung zur Projektverantwortlichkeit. Die Umsetzung der Maßnahmen sollte zeitnah erfolgen, um die Arbeitssituation in absehbarer Zukunft zu verbessern.

Die einzelnen Schritte für den Ablaufplan einer Arbeitssituationsanalyse werden abschließend mithilfe von Tab. 2 zusammengefasst.

**Table 2:** 

Schritt	Inhalt	Zeit
1. Den Rahmen schaffen	Dauer und Zeitrahmen der ASITA festlegen	–
	Teilnehmer*innen einladen	
	Moderation benennen	
	Raum buchen und Arbeitsmaterialien anfordern	
2. Einen gelungenen Einstieg finden	Vorstellungsrunde	10 min ∑ = 10 min
	Ablauf und Ziel der ASITA erläutern	
	Regeln der Zusammenarbeit erklären	
3. Stärken und Ressourcen erfassen	Stärken und Ressourcen der Arbeitssituation werden erfasst und auf Karten geschrieben	10 min ∑ = 20 min
4. Veränderungsbedarf messen	Veränderungsbedarf in der Arbeitssituation einschätzen	5 min ∑ = 25 min
	Teilnehmer*innen kleben Punkte	
	Moderator*innen werten aus	
5. Veränderungsbedarfe vorstellen	Moderation stellt Veränderungsbedarfe und beispielhafte Themen vor	10 min ∑ = 35 Min
6. Veränderungsbedarfe identifizieren und priorisieren	Veränderungsbedarfe vorstellen und priorisieren	5 min ∑ = 40 Min
	Teilnehmer*innen kleben Punkte an die Bereiche, die sie am wichtigsten empfinden	
7. Problemfelder konkretisieren und Lösungen erarbeiten	Problemfelder konkretisieren und Lösungen erarbeiten	60 min ∑ = 100 Min
	Moderator*innen leiten eine Diskussionsrunde mit den Teilnehmern	
8. Ergebnisse freigeben	Ergebnisse zusammenfassen	10 min ∑ = 110 min
	Absegnung zur Freigabe der Ergebnisse einholen	
9. Einen gelungenen Abschluss finden	Moderation gibt kurzen Ausblick auf den weiteren Prozess	5 min ∑ = 115 min
	Moderator*innen bedanken und verabschieden sich	
10. Einen Ergebnisbericht schreiben	Ergebnisse in einem Bericht dokumentieren	–
	Fotoprotokoll einschließlich der Fotos von den Metaplanwänden und Flipcharts erstellen	
	Ergebnisse an die Führungsebene kommunizieren	

Quelle: eigene Darstellung in Anlehnung an: BGN [2]

## Anpassung an das digitale Format

Aufgrund der Corona-Situation war eine Umsetzung der Arbeitssituationsanalysen für das Gesundheitsprojekt in der Einrichtung vor Ort nicht möglich. Alternativ wurde die Analyse in einem Onlineformat durchgeführt. Für das Projekt wurde das Produkt *Skype for Business* als Kommunikations- und Videokonferenz-Tool verwendet, sodass die Teilnehmer*innen über die Funktion des geteilten Bildschirms durch eine PowerPoint-Präsentation geführt werden konnten. Als digitales Abstimmungstool kam die Plattform *PINGO* zum Einsatz, mit der sowohl offene als auch geschlossene Fragen beantwortet werden konnten. PINGO steht für „peer instructions for very large groups“ und wurde in einer Kooperation mit der Universität Paderborn als ein webbasiertes Live-Feedback-System entwickelt [18]. Es bietet aufgrund der Server-Standorte in Deutschland eine datenschutzkonforme und sichere Anwendung. Die Fragen werden stets einzeln und nicht in Form einer komplexen Umfrage gestellt, damit die Fortführung der Gruppendiskussion von der Moderation beeinflusst werden kann [18]. Um an den Umfragen teilnehmen zu können, wurde ein Link zur Weiterleitung an das Abstimmungstool über die Chatfunktion von Skype for Business geteilt.

Nach einer ersten Begrüßung wurden die Zielsetzung der Befragung und die Regeln der Zusammenarbeit vermittelt. Besonderen Wert wurde dabei auf die Zusicherung der Vertraulichkeit gelegt und auf die Notwendigkeit der aktiven Mitarbeit. Weiterhin werden die Teilnehmer*innen mit PINGO vertraut gemacht.

Für die Feststellung der *Ist-Situation* wurde eine quantitative Evaluation durch die Bepunktung der verschiedenen Bereiche der Arbeitssituation durchgeführt. Dies wurde über das Abstimmungstool PINGO realisiert. Die 7 zu untersuchenden Bereiche sind das Arbeitsumfeld, Arbeitsmittel, Führung, Arbeitsorganisation, Arbeitsaufgabe, Beziehung zu Kolleg*innen und *Sonstiges* und weichen damit minimal von den in Tab. 1 erläuterten Bereichen ab. Die Bereiche Arbeitsumfeld und Arbeitsorganisation bleiben unverändert, während die Arbeitsaufgabe mit der Arbeitstätigkeit gleichgesetzt wird. Beim Arbeitsumfeld soll beispielsweise untersucht werden, inwiefern es Störfaktoren am Arbeitsplatz im Homeoffice gibt und wie das Arbeitsumfeld insgesamt empfunden wird. Der Bereich Arbeitsorganisation umfasst sämtliche Faktoren, die zur Planung der Arbeitsstruktur sowie zur Kommunikation und Abstimmung im Team gehören. Zum Beispiel sollen die Fragen „Wie werden die Tage im Homeoffice und die Arbeitszeiten vor Ort organisiert?“ und „Welche Regelungen gibt es für Urlaubstage?“ beantwortet werden. Die Arbeitsaufgabe fragt nach der Arbeitstätigkeit und ob diese sich im Homeoffice stark verändert hat. Zudem wird beleuchtet, ob es ein Weiterbildungsangebot gibt sowie Unterstützung für die Erfüllung der Arbeitsaufgaben. Das Gruppen- bzw. Betriebsklima wird durch den Bereich Beziehung zu Kolleg*innen ersetzt, da durch die Arbeit auf Distanz insbesondere die Zusammenarbeit und die Kommunikation mit den Kolleg*innen und dementsprechend die Beziehungen beeinflusst werden. Veränderungen im Gruppen- und Betriebsklima können Folgen dieser Beziehungen zu Kolleg*innen sein. Zusätzlich wurde die Arbeitssituation um den Bereich Arbeitsmittel ergänzt, denn ein produktives und effizientes Arbeiten im Homeoffice ist abhängig von den Rahmenbedingungen des Heimarbeitsplatzes und erfordert eine entsprechende technische Ausstattung. Welche Arbeitsmittel stehen zur Verfügung, und werden diese vom Unternehmen bereitgestellt? Für den Bereich Führung ist zu beachten, dass für die Gruppe 1 und 2 (Mitarbeiterebene) zunächst auf die direkte Führung bzw. die direkten Vorgesetzten eingegangen werden soll, nicht auf die Geschäftsführung. Die Erweiterung um den Bereich Sonstiges ist wichtig, um Ergänzungen zu Themen aufzugreifen, die sich den anderen Bereichen nicht zuordnen lassen und somit nicht abgedeckt werden.

Jeder der 7 Bereiche wird nacheinander durch die Nutzung des digitalen Abstimmungstools mit der Frage „Wie bewerte ich den Bereich …?“ bewertet. Die Teilnehmenden müssen sich eindeutig für eine der 4 Antwortmöglichkeiten („zufrieden“, „teils zufrieden“, „teils unzufrieden“, „unzufrieden“ und „keine Anmerkung“) entscheiden.

Die Antwortmöglichkeit „keine Anmerkung“ steht dabei nur für den Bereich Sonstiges zur Verfügung. Nach jeder Abstimmung erfolgt eine kleine Auswertung, und zum Schluss wird das Gesamtstimmungsbild dargestellt.

**Figure 1:**
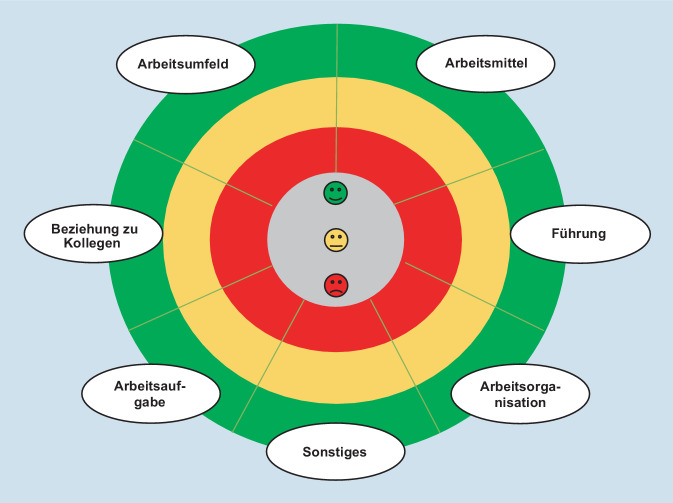

Die Darstellung des Gesamtstimmungsbildes und die *Auswertung* der Ist-Analyse erfolgt durch eine Bewertung der Ergebnisse nach einem Ampelsystem. Die Farbe Grün steht für „zufrieden“, Gelb für „teils zufrieden, teils unzufrieden“ und Rot für „unzufrieden“. Veranschaulicht wird das Stimmungsbild mithilfe der *Arbeitssituations-Spinne*. Diese durch die Moderation genutzte Methode der Arbeitssituations-Spinne wird in Abb. 1 demonstriert.

Im nächsten Schritt wird eine *Konkretisierung* der Ergebnisse durchgeführt bzw. das Stimmungsbild der Arbeitssituations-Spinne qualitativ evaluiert. Dieser Prozess beinhaltet, dass zu den Schwerpunkten aus der quantitativen Analyse Themencluster durch Befragung der Teilnehmenden gebildet werden. Zunächst wird mündlich die Frage gestellt, woran die Teilnehmer*innen bei dem Bereich Sonstiges gedacht haben. Anschließend wird bewertet, inwieweit eine Veränderung der Arbeitssituation und in welchen Bereichen diese Veränderung für wichtig empfunden wird. Die Ergebnisse zeigen, in welchen Bereichen Zufriedenheit oder Unzufriedenheit besteht. Die Bereiche mit der meisten Kritik werden in den Fokus genommen und anhand der Frage „An was haben Sie gedacht, als Sie den Bereich … bepunktet haben?“ untersucht. Die Moderation leitet die Gruppendiskussion: Problemfelder sowie erste Lösungsansätze werden erarbeitet und für alle Teilnehmer*innen sicht- und nachvollziehbar auf den entsprechenden Folien einer PowerPoint-Präsentation protokolliert.

Die *Ideensammlung* wurde mithilfe der Vier-Felder-Methode zu einem durch die Moderation und die Teilnehmer*innen gemeinsam festgelegten Thema durchgeführt. Das zu diskutierende Thema basiert auf einem Bereich, dessen Verbesserung im Interesse der Teilnehmer*innen und des Unternehmens liegt. Die Ideen werden für die 4 Felder *Problem und Auswirkung, Ziel in einem Jahr, Hindernisse* und *nächste Schritte* gesammelt. Ein Grundriss dieser Ideensammlung durch die Vier-Felder-Methode lässt sich in Tab. 3 erkennen.

**Table 3:** 

Thema	
*Problem → Auswirkung*	*Ziel in einem Jahr*
Problem und Auswirkung 1	Ziel 1
Problem und Auswirkung 2	Ziel 2
…	…
*Hindernisse*	*Nächste Schritte*
Hindernis 1	Schritt 1
Hindernis 2	Schritt 2
…	…

Der abschließende Teil der Arbeitssituationsanalyse beinhaltet eine *Zusammenfassung* der Ergebnisse und Erkenntnisse. Zudem wird geklärt, welche Folien und Notizen in das Fotoprotokoll aufgenommen werden. Die Moderation gibt einen Ausblick auf das zukünftige Vorgehen sowie die weiteren Schritte.

Nach den Arbeitssituationsanalysen werden die Protokolle der verschiedenen Gruppen der öffentlichen Verwaltung verteilt. Während des Auswertungstermins stellt die Moderation der Geschäftsführung die gesundheitsrelevanten Bedrohungen und Ressourcen vor. Sie beschreibt deren Zusammenhänge sowie die in den Gruppendiskussionen gemachten Beobachtungen. Weiterhin werden Maßnahmenideen und Empfehlungen für eine Verbesserung der zukünftigen Arbeitssituation abgeleitet. Vereinbarungen zum weiteren Vorgehen werden getroffen. Der Stand der Maßnahmenumsetzung wird mitverfolgt. Für die erfolgreiche Umsetzung der Lösungsstrategien werden die Beschäftigten zeitnah und regelmäßig über den aktuellen Stand und die weiteren Entwicklungsschritte informiert.

## Ergebnisse

### Arbeitssituationsanalysen auf Mitarbeiterebene

Die Arbeitssituationsanalysen der ersten beiden Gruppen aus der Mitarbeiterebene werden aufgrund einer geringeren Teilnehmerzahl (Gruppe 1 *n* = 7, Gruppe 2 *n* = 3) als geplant zusammengefasst, um den Datenschutz entsprechend gewährleisten zu können. An den Befragungen und Diskussionen haben sowohl weibliche (*n* = 8) als auch männliche Mitarbeiter*innen (*n* = 2) teilgenommen, die im Durchschnitt einen bis 5 Tage die Woche im Homeoffice arbeiten. Der Kundenkontakt variiert je nach Tätigkeitsbereich von hoch bis eher niedrig.

**Figure 2:**
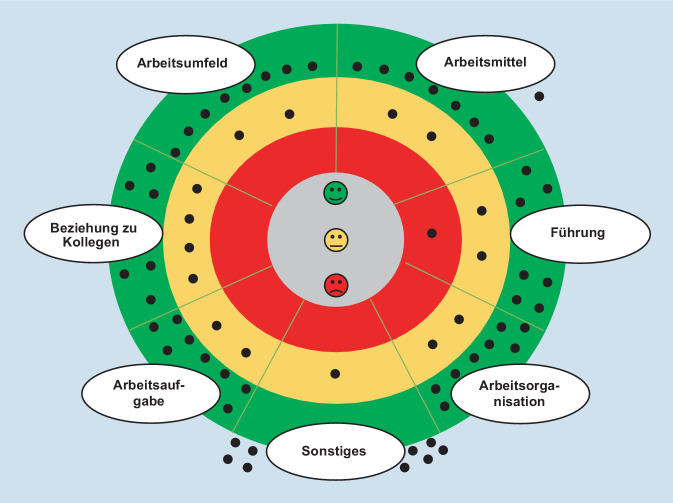

Die Frage, wie die 7 Bereiche der Arbeitssituation (Arbeitsumfeld, Arbeitsmittel, Führung, Arbeitsorganisation, Arbeitsaufgabe, Beziehung zu Kolleg*innen und Sonstiges) von den Mitarbeiter*innen empfunden und bewertet werden, ergab das in Abb. 2 zu erkennende Stimmungsbild.

Die Punkte im grünen Bereich spiegeln ein positives Empfinden wider, während sie im gelben Bereich für zum Teil zufrieden/zum Teil unzufrieden und im roten Bereich für Unzufriedenheit stehen. Enthaltungen werden durch die Punkte außerhalb der 3 Zonen gekennzeichnet. Grundsätzlich lässt sich ein durchaus positives Empfinden seitens der Mitarbeiter*innen hinsichtlich ihrer Arbeitssituation entnehmen. Jeweils 8 von 10 Teilnehmer*innen geben an, in den Bereichen Arbeitsaufgabe, Arbeitsorganisation und Arbeitsumfeld zufrieden zu sein, während zwei Mitarbeiter*innen teils zufrieden/teils unzufrieden sind. Im Bereich Arbeitsmittel sind 7 der 10 Mitarbeiter*innen zufrieden und 2 Mitarbeiter*innen teils zufrieden/teils unzufrieden. Es gibt eine Enthaltung. Leichte Tendenzen zu einer Unzufriedenheit lassen sich insbesondere in den Bereichen Führung und Beziehung zu den Kolleg*innen festhalten. Vier der 10 Befragten sind zum Teil zufrieden/zum Teil unzufrieden hinsichtlich ihrer Beziehung mit den Kollegen. In Bezug auf die direkte Führung gibt ein Teilnehmer an, unzufrieden zu sein und zwei weitere Teilnehmer*innen stimmen für den gelben Bereich. Es gibt jedoch keinen Bereich, der sich komplett in der roten Zone befindet. Ein Großteil der Befragten hat keine Anmerkungen zum Bereich Sonstiges. Im Folgenden werden die Ergebnisse der Ist-Analyse und das Stimmungsbild der Arbeitssituation-Spinne ausgewertet und analysiert, wobei verstärkt untersucht wird, an was die Befragten bei dem Gedanken an die Bereiche Führung und Beziehung zu Kollegen gedacht haben.

### Arbeitsmittel und -umfeld

Die Mitarbeiter*innen sind im Großen und Ganzen mit ihren Arbeitsmitteln im Homeoffice sehr zufrieden. Teilweise ist der Heimarbeitsplatz besser ausgestattet als im Büro mit beispielsweise zwei Bildschirmen. Diese technische Ausstattung zu Hause ist jedoch in den meisten Fällen von einem hohen individuellen Engagement abhängig. Anschaffungen werden selbstständig und auf eigene Kosten getätigt, um das Homeoffice den persönlichen Wünschen und Anforderungen entsprechend einzurichten. Neben dem Zeitaufwand wird es als schwierig und umständlich empfunden, die Technik am betrieblichen Arbeitsplatz wiederholt auf- und abbauen zu müssen, um diese nach Hause zu transportieren. Problematisch kann dies in Hinblick auf spezifische Vorschriften, wie z. B. die Verschlüsselung von E‑Mails werden, da die Technik daheim nicht die erforderlichen Funktionen für den Einsatz der elektronischen Dienstkarte besitzt und somit trotzdem die Tastatur aus dem Büro mitgenommen werden muss. Allerdings ist es nicht immer möglich, die gesamte Technik mit nach Hause zu nehmen, da es Präsenztage gibt, an denen mehrmals die Woche im Betrieb den Arbeitsaufgaben nachgegangen werden muss. Dementsprechend gibt es Mitarbeiter*innen, denen im Homeoffice nur ein Laptop zur Verfügung steht. Es wird bemängelt, dass es keinen technischen Standard für zu Hause gibt. Ferner stellt sich die Frage, inwiefern die Bürotechnik zu Hause versichert ist. Dies stellt eine erhebliche Barriere beim mobilen Arbeiten dar. Weiterhin kann die eigene Internetverbindung die Arbeit im Homeoffice behindern, wenn die Internetleitung nicht stabil genug ist. Beim Wechsel ins Homeoffice zu Beginn der SARS-CoV-2-Pandemie wurden die Mitarbeiter*innen mit vielen Themen und Problemen allein gelassen. Erklärungen und Informationen gab es erst auf Nachfrage. Eine Unterstützung beim Einrichten des Homeoffice-Arbeitsplatzes ist wünschenswert. Ferner muss beachtet werden, dass es aufgrund der vielen verschiedenen technischen Ausstattungen für die IT-Abteilung schwierig ist, die entsprechenden Informationen zu kommunizieren.

In Hinblick auf das Arbeitsumfeld ist ein Großteil der Befragten mit dem Arbeitsplatz zu Hause zufriedener als im Büro. Zudem sind die Mitarbeiter*innen motiviert, das Arbeitsumfeld nach ihren eigenen Bedürfnissen zu gestalten. Das Umfeld im Büro ist mit einem höheren Lärmpegel und mehr Ablenkung verbunden, während es nach Ansicht der Teilnehmer*innen zu Hause weniger Störfaktoren, mehr Ruhe für ein konzentriertes und produktives Arbeiten gibt. Beispielsweise entfällt das Teilen eines Büros mit weiteren Arbeitskolleg*innen, sodass z. B. zeitgleiches Telefonieren oder parallele Besprechungen im Büro entfallen. Viele Mitarbeiter*innen geben jedoch an, keine Kinder zu Hause zu haben, die betreut werden müssen, da diese in der Kita oder Schule beaufsichtigt werden. Außerdem lernen die Kinder, die Eltern bei der Arbeit nur in Notfällen zu unterbrechen. Bedauerlicherweise gibt es Mitarbeiter*innen, die zu Hause kein separates Arbeitszimmer haben, sodass die Arbeit am Wohnzimmer- oder Küchentisch erledigt wird. In diesen Fällen existiert kein richtiges Arbeitsumfeld, kein ergonomischer Arbeitsplatz und die Mitarbeiter*innen sind wiederum von ihrer Eigeninitiative abhängig.

### Arbeitsaufgabe und -organisation

Die Mitarbeiter*innen empfinden durch die Corona-Krise und die damit einhergehende Arbeit im Homeoffice einen starken Wandel der Arbeitsaufgabe und -organisation. Neue Chancen, aber auch Herausforderungen sind entstanden. Vor der Pandemie war die Arbeitsaufgabe und -organisation zum Teil durch einen starken Kundenkontakt und auch zahlreiche persönliche Kontakte geprägt, während die Kontaktbeschränkungen, Abstandsregeln und Hygienevorschriften zu neuen Erfahrungen führen. Es stellt sich die Frage, inwieweit die täglichen persönlichen Kontakte einen Mehrwert bringen und ob die veränderte Arbeitsorganisation und -struktur die Beschäftigten und Kunden hinsichtlich ihrer Motivation und Zufriedenheit negativ beeinflussen. Viele Kund*innen nehmen die Telefontermine beispielsweise gerne an. Es zeigt sich ein gewisser Erziehungseffekt, dass Kund*innen auch den telefonischen Weg wählen. Von Vorteil ist zudem, dass die Kund*innen nicht „für jede Kleinigkeit“ einen Termin vereinbaren und eine schnellere Problemlösung möglich ist. Allerdings ist es durchaus möglich, Kund*innen zu verlieren, die sich telefonisch nicht erreichen lassen. Diese Kund*innen sind aber in den meisten Fällen auch für Präsenztermine schwer zu erreichen. Der Weg der Digitalisierung wird als besonders schwierig empfunden. Die Arbeitsprozesse sollten vereinfacht werden. Noch immer gibt es sehr viel Papierpost, die die am betrieblichen Arbeitsplatz verbliebenen Kolleg*innen zusätzlich zu den eigenen Aufgaben erledigen müssen. Dadurch entstehen häufig Konflikte mit den Arbeitskolleg*innen im Homeoffice. Es muss berücksichtigt werden, dass nicht jede*r Mitarbeiter*in im Homeoffice arbeiten möchte und somit eine Ungleichbehandlung entstehen kann. Hinsichtlich der Arbeitsorganisation merken die Mitarbeiter*innen an, dass sie sich durch die Arbeit im Homeoffice besser organisieren und aufgrund reduzierter Ablenkungen und Unterbrechungen die Arbeitsaufgaben effektiver erledigen können. Im Zusammenhang mit einer effektiveren Arbeitsorganisation steht auch ein verbessertes Gesundheitsempfinden. Durch die Arbeit im Homeoffice entfallen Fahrtwege und -zeiten, was zu einem Gewinn an mehr Freizeit und Entspannung führt. Es geht keine wertvolle Zeit im Auto oder in der Straßenbahn auf dem Weg ins Büro verloren, sodass weniger Druck auf den Mitarbeiter*innen lastet und andere Aufgaben, z. B. in der Familie, besser erfüllt werden können. Die Befragten fühlen sich gesünder und freuen sich zudem über eine geringere Umweltbelastung durch ein niedrigeres Verkehrsaufkommen.

### Beziehung zu den Kolleg*innen

Die SARS-CoV-2-Pandemie hat die Kooperation zwischen den Kolleg*innen stark verändert, aber es wird insgesamt in vielen Bereichen ein gutes Teamgefüge wahrgenommen. Durch die Arbeit auf Distanz musste ein neues Miteinander gefunden werden. Es hat eine gewisse Zeit in Anspruch genommen, sich in der neuen Situation einzufinden. Insbesondere die sozialen Aspekte der Kommunikation fallen weg. Zudem reduziert sich durch die Arbeit im Homeoffice der Kontakt zu den Kolleg*innen. Einige Ansprechpartner werden bevorzugt, während der Kontakt zu anderen Kolleg*innen abnimmt. Die elektronische Akte erleichtert den Austausch und die Verwaltung relevanter Informationen und Daten, dennoch ersetzt auch sie die sozialen Aspekte der Kommunikation nicht. Zudem führt die Tatsache, dass Homeoffice nicht bei allen Kolleg*innen anerkannt und akzeptiert wird, zu zahlreichen Konflikten. Es kommt zu Diskussionen und Vorurteilen, die die Effektivität und Produktivität der Arbeit im Homeoffice anzweifeln. Beispiele für derartige Kommentare sind: Während der Arbeitszeit wird zu Hause gekocht, gepuzzelt oder Wäsche gewaschen. Das Positive am Homeoffice wird von einigen Kolleg*innen nicht gesehen und bewusst ignoriert. Eine abwertende Meinung solcher Kolleg*innen bezüglich der Arbeit zu Hause hat letztendlich eine größere Zurückhaltung bei der Inanspruchnahme von Homeoffice zur Folge.

### Führung

Zum einen wird die Arbeit mit der direkten Teamleitung und den Bereichsleitern größtenteils als sehr positiv empfunden, während zum anderen auch im Bereich der Führungskräfte das Problem der Anerkennung und Akzeptanz der Arbeit von zu Hause zum Ausdruck kommt. Die Mitarbeiter*innen erläutern, dass der Wechsel der Arbeitsweisen von Präsenzarbeit zur Homeoffice-Arbeit gut geglückt ist. Am Anfang der Pandemie gab es noch Unsicherheiten, aber diese wurden schnell aus dem Weg geräumt. Insbesondere die Kommunikation und Unterstützung der direkten Führungskräfte wird gelobt. Es gibt regelmäßige Telefonate, E‑Mails und Vor-Ort-Meetings, um die anstehenden Arbeitsaufgaben zu besprechen, sich zu erkundigen, wie es den Mitarbeiter*innen geht und ob es Probleme gibt. Beispielsweise wird auch abgestimmt, wer als Notfallteam an welchen Tagen im Betrieb sein muss. Auch die Führungskräfte werden im Umgang mit Homeoffice vor neue Herausforderungen gestellt und müssen sich dieser neuen Situation anpassen. Die veränderte Kommunikation führt zu Misstrauen und einem Kontrollverlust. Die Mitarbeiter*innen warnen vor einer übermäßigen Anzahl von Telefonanrufen durch den Chef oder die Chefin. Während vor Ort eine direkte, sofortige Kontaktaufnahme möglich war, muss diese im Homeoffice auf eine neue Art und Weise praktiziert werden. Vonseiten der Geschäftsführung wird sich mehr Präsenz gewünscht, um Unternehmenskultur weiterzuentwickeln. Der direkte Kontakt beispielsweise über kurze Flurgespräche ist ein erster Schritt, um eine neue Verbindung aufzubauen. Nach einer Stellungnahme der Geschäftsführung ist weiterer Gesprächsbedarf zum Thema Homeoffice signalisiert worden. Zu Beginn der Pandemie liefen die Prozesse schleppend an und es gab keine schnellen Entscheidungen. Homeoffice wurde weder abgelehnt noch gefördert, und Veränderungen wurden erst mit der Homeoffice-Pflicht der SARS-CoV-2-Arbeitsschutzverordnung eingeleitet. Das Unternehmensbild zum Homeoffice ist wichtig, um das Vertrauensproblem der Führungsebene hinsichtlich der Arbeit der Mitarbeiter*innen im Homeoffice zu korrigieren. Ohne die entsprechende Zustimmung und Akzeptanz der Führung haben die Mitarbeiter*innen zum Teil ein schlechtes Gewissen, die Arbeit von zu Hause in Anspruch zu nehmen.

### Arbeitssituationsanalyse der Bereichsleiterebene

**Figure 3:**
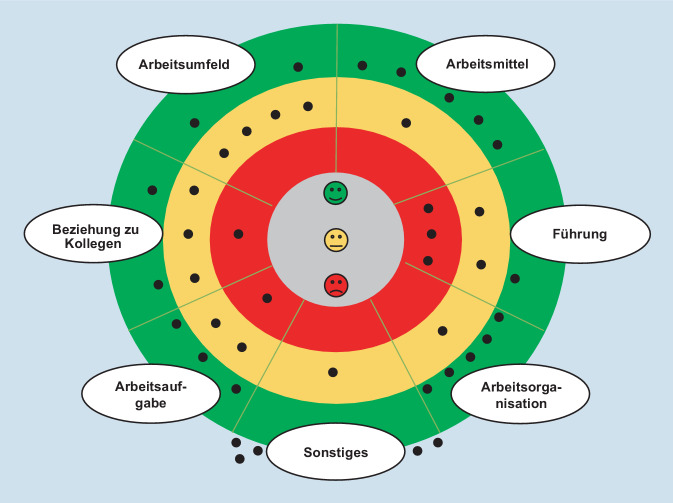

Die Arbeitssituationsanalyse der 3. Gruppe setzt sich aus weiblichen (*n* = 2) und männlichen (*n* = 4) Bereichsleiter*innen zusammen. Die Arbeit im Homeoffice ist bei den insgesamt 6 teilnehmenden Führungskräften mit gar keinen oder bis zu zweieinhalb Tagen im Homeoffice weniger präsent als bei den Mitarbeiter*innen. Das Stimmungsbild der Bereichsleiter*innen fällt grundsätzlich kritischer aus als das der Mitarbeiter*innen. In allen Bereichen geben die Führungskräfte an, nur zum Teil zufrieden zu sein. In den Bereichen Beziehung zu Kolleg*innen und Arbeitsaufgabe gibt jeweils ein*e Teilnehmer*in der Gruppe an unzufrieden zu sein, während im Bereich Führung 3 der 6 Befragten, und damit die Hälfte der Teilnehmer*innen, für die rote Zone stimmen, also unzufrieden sind. Als besonders positiv werden die Arbeitsmittel und die Arbeitsorganisation empfunden. Jeweils 5 der 6 Teilnehmer*innen finden sich im grünen Bereich wieder und ein*e Teilnehmer*in im gelben Bereich. Die Auswertung der quantitativen Befragung und das Gesamtstimmungsbild werden in Abb. 3 verdeutlicht.

### Arbeitsmittel und -organisation

Nach Einschätzung der Führungskräfte stehen ihnen alle notwendigen Arbeitsmittel, vor allem am betrieblichen Arbeitsplatz, zur Verfügung. Die Ausstattung ist oftmals neuwertig, und ergonomische Arbeitsmittel, wie z. B. Stehtische, können beantragt werden. Allerdings wird von den Bereichsleitern kritisch angemerkt, dass die Mitnahme von technischen Geräten ins Homeoffice einen zusätzlichen Zeit- und Stressfaktor darstellt. Zum Beispiel kann der PC zu Hause nicht mit der elektronischen Dienstkarte genutzt werden, sodass die Tastatur aus dem Büro mitgenommen werden muss. Alternativ konnte in der Verwaltungseinrichtung ein Card Reader bestellt werden, der jedoch aufgrund der erhöhten Nachfrage während der SARS-CoV-2-Pandemie aktuell nicht mehr zur Verfügung steht. Eine weitere Möglichkeit besteht darin, sich eigenständig einen Card Reader zuzulegen. Damit kommt nicht nur bei den Mitarbeiter*innen, sondern auch bei den Führungskräften das Thema der Eigeninitiative zum Ausdruck. Zusammengefasst ist das Thema der technischen Ausstattung nicht vollends ausgereift und es muss vonseiten der Geschäftsführung nachgesteuert werden. Insgesamt ist aber durch eigene Kompetenzen eine gute Arbeitsorganisation möglich.

### Arbeitsumfeld

Hinsichtlich des Arbeitsumfeldes gibt es durchaus geteilte Meinungen, die in engem Zusammenhang mit der SARS-CoV-2-Arbeitsschutzverordnung stehen. Einerseits leidet aufgrund der Arbeitsschutzverordnung insbesondere das Arbeitsumfeld in Bereichen des Unternehmens, wo nur Gruppen- oder Doppelbüros zur Verfügung stehen. Andererseits wird das veränderte Arbeitsumfeld in anderen Bereichen des Unternehmens als sehr angenehm empfunden, da sich beispielsweise der Kundenstrom reduziert hat und die erforderlichen Einzelbüros in anderen Bereichen bereits zur Verfügung stehen. Die Frage, wie alle Kolleg*innen mit einer Einzelplatzlösung versorgt werden, führt zu massiven organisatorischen Anforderungen, denn nur eine begrenzte Anzahl an Mitarbeiter*innen darf zusammen in einem Raum sitzen. Insgesamt gibt es im Unternehmen zu wenige Räumlichkeiten für alle Mitarbeiter*innen, sodass ein wöchentlicher Bürowechsel entstanden ist. Phasenweise gibt es Kolleg*innen, die dauerhaft im Homeoffice bleiben müssen, um die gesetzlichen Rahmenbedingungen abdecken zu können. Auch hier ist es wichtig anzumerken, dass die Mitnahme von Spezialausstattungen ins Homeoffice zu Komplikationen in Bezug auf die Inbetriebnahme und Nutzung führen kann. Insgesamt ist aber eine Einsicht zur Notwendigkeit dieser Maßnahmen vorhanden.

### Beziehung zu Kolleg*innen

Nach Aussagen der Führungskräfte hat die SARS-CoV-2-Pandemie die sozialen Aspekte der Zusammenarbeit und den Teamgeist stark beeinflusst. Das kollegiale Miteinander wird eingeschränkt, da die Kolleg*innen am betrieblichen Arbeitsplatz weniger präsent sind und damit nicht so häufig gesehen werden. Insbesondere Kolleg*innen mit Kindern arbeiten bis zu 5 Tage die Woche im Homeoffice. Mit einem Großteil der Beschäftigten, der von zu Hause arbeitet, ist es schwieriger, eine gemeinsame Aufgabe, ein gemeinsames Ziel sowie eine gemeinsame Sichtweise zu verfolgen. Skype- und Telefonabsprachen können Mimik und Gestik nicht ersetzen, und es lässt sich oftmals nicht erkennen, ob die Kolleg*innen Verständnis für die besprochenen Arbeitsthemen haben oder eine ablehnende Haltung einnehmen. Es besteht die Gefahr, dass die reduzierten Kontakte mit den Kolleg*innen und damit ein geringer Austausch sowie eine schlechte Zusammenarbeit schnell zur Normalität werden. Der Verlust der Zwischenmenschlichkeit geht zudem mit einem erhöhten Leistungsdruck einher, es fällt zum Teil schwer, Ruhepausen einzuhalten. Unterhaltungen mit Kolleg*innen am Dienstort entfallen. Es entsteht ein Pflichtgefühl, das Telefon zu Hause ständig mitzuführen, z. B. wenn ein kurzer Toilettengang von Nöten ist. Eine große Herausforderung und mehr Arbeitsaufwand werden auch in der Einarbeitung neuer Kolleg*innen über digitale Arbeitsmittel und das Homeoffice gesehen. Die Führungskräfte betonen außerdem ihren Wunsch und die Notwendigkeit, die gegenseitige Toleranz für das Arbeiten in Präsenz und im Homeoffice zu fördern. Vorurteile hinsichtlich der Arbeitsproduktivität zu Hause haben einen negativen Einfluss auf das Teamgefüge und führen zu Konflikten.

### Arbeitsaufgabe

Im Fokus der Diskussionen mit den Führungskräften stand vor allem das Thema der Arbeitsaufgabe, da in diesem Bereich ein starker Wandel durch die SARS-CoV-2-Pandemie wahrgenommen wird. Drei Probleme wurden hervorgehoben. Das *erste Problem* ist eine faire Aufgabenverteilung. Die Führungskräfte empfinden eine ungleiche Verteilung der Aufgaben und eine Zunahme an Zusatzaufgaben. Diese „On-Top-Aufgaben“ erhöhen das Stresserleben, da es keine Entlastung in anderen Aufgabenbereichen gibt, um der Überlastung entgegenzuwirken. Eng damit verbunden ist das* zweite Problem*, das in einer starken Änderung der Führungsaufgabe gesehen wird. Es müssen vollständig andere Aufgaben erledigt werden, die vor der Pandemie nicht existent waren. Dazu zählen insbesondere Managementaufgaben von Beschäftigten im Homeoffice oder in Präsenz und die damit einhergehende digitale Kommunikation. Die Informationsvermittlung und Kommunikation sind ein schwieriges Thema, denn eine rein schriftliche Kommunikation, z. B. über E‑Mail, ist nicht geeignet, den direkten Kontakt zu den Mitarbeiter*innen herzustellen und das nötige Feedback zu den Arbeitsthemen zu erhalten. Die Führungskräfte müssen sich mit zahlreichen neuen Fragen auseinandersetzen, wie z. B. „Wie kommuniziere ich mit meinem Team?“, „Wie halte ich das Team zusammen?“, „Welche Besprechungsformate nutze ich, um die Mitarbeiter zu erreichen?“, „Wie gehe ich auf Kolleg*innen zu, um Fachliches auszuwerten?“ und „Wer sitzt wo, wie und wann?“. Diese Fragen sind mit einer Vorteilsübersetzung verbunden, d. h. warum die Aufgaben in der vorgeschriebenen Art und Weise erfüllt werden müssen. Die Arbeitsorganisation vonseiten der Führungskräfte hat an Bedeutung gewonnen, da mehr Termine untergebracht werden müssen und ein höheres Maß an Selbstorganisation erforderlich wird. Zudem gilt es zu klären, wie die Mitarbeiter*innen der Teams, die vor allem durch einen hohen Kundenkontakt geprägt sind, nach der Pandemie wieder in die Präsenzarbeit zurückgeführt werden können. Aufgrund dieser anspruchsvollen Aufgaben werden von den Führungskräften vermehrte Stressmomente wahrgenommen. Die Änderung der Führungsaufgaben steht zudem in Zusammenhang mit dem dritten Problem. Das *dritte Problem* beschäftigt sich mit der Nutzung von Skype for Business oder ähnlichen virtuellen Kollaborationstools. Einerseits ist es über Skype einfacher, sich von einer Veranstaltung in die nächste einzuwählen, aber andererseits ist es auch schwieriger vom Homeoffice den Kontakt zu den Mitarbeiter*innen zu halten und wissensmäßig auf dem neuesten Stand zu sein. Weiterhin nimmt die Nutzung von Skype vermehrt zu und wird für viele nicht dienstliche Zwecke missbraucht. Im Homeoffice können alltägliche Unterbrechungen durch Kolleg*innen leichter unterbunden werden, um beispielsweise eine E‑Mail zu Ende zu schreiben. Seitdem Skype im Unternehmen genutzt wird, ist die Beratungslast und die Anzahl an Besprechungen, insbesondere in der Führungsebene, exorbitant gestiegen. Selbst für eine kurze Nach- und Zwischenfrage wird ein Skype-Termin eingeplant. Die Kommunikation wird folglich in viele kleine Einzelthemen zersplittert. Skype wird damit zu einem zeitlichen Problem und Störfaktor sowohl für die Beschäftigten im Büro als auch im Homeoffice. Ständige Besprechungen für Geringfügigkeiten unterbrechen die täglichen Arbeitsaufgaben und lassen keine Zeit für die eigentliche, originäre Arbeit. Grundsätzlich wird Skype als eine positive Plattform für die Kommunikation und Arbeit gesehen, aber die Masse an Skype Terminen wird als Belastung empfunden.

### Führung

Die Führungskräfte teilen die Meinung der Mitarbeiter*innen in Bezug auf die Geschäftsführung und äußern den Wunsch einer Stellungnahme der Geschäftsführung zum Thema Homeoffice. Es gibt keine Orientierung und keinen Leitfaden für das Arbeiten im Homeoffice, sodass sich die Bereichsleiter in ihren Entscheidungen zum Teil allein gelassen und hilflos fühlen. Beispielsweise müssen sie begründen, warum nicht alle Mitarbeiter*innen die Erlaubnis für die Arbeit von zu Hause bekommen. Es gibt keine konkreten Vorgaben, wonach z. B. ein*e Mitarbeiter*in mehr als drei Tage Homeoffice pro Woche in Anspruch nehmen darf. Dies beeinträchtigt die Führungskräfte in ihren Tätigkeiten. Für den Unternehmenserfolg ist es wichtig, dass nicht jeder Bereich seine eigenen Regeln aufstellt und Entscheidungen trifft. Die fehlende Präsenz des Geschäftsführers wird bemängelt. Diese hat sich insbesondere zu Beginn der Pandemie durch eine fehlende Unterstützung der Bereichs- und Teamleiter*innen bemerkbar gemacht. Weiterhin erschwert die Abwesenheit und das fehlende positive als auch negative Feedback den Zusammenhalt und die Zusammenarbeit im Unternehmen. Das Feedback, auch in Form von Kritik, ist für eine Weiterentwicklung der öffentlichen Verwaltungseinrichtung unverzichtbar. Die mangelnde Wertschätzung hat unterdessen eine Unzufriedenheit der Führungskräfte zur Folge. Durch die Arbeit auf Distanz ist eine Wertschätzung und Kommunikation auf Augenhöhe noch schwieriger, da kurze Gespräche mit den Vorgesetzten im Unternehmen meist gänzlich entfallen.

## Übersicht der Ergebnisse

Die Auswertung der Gruppendiskussionen zeigt, dass es durchaus Potenzial für die Verbesserung der Arbeitssituation und für die Umsetzung und Gestaltung von Homeoffice im Betrieb gibt. Tab. 4 bietet eine abschließende Zusammenfassung der positiven und negativen Einflussfaktoren der einzelnen Arbeitsbereiche aus Sicht der Mitarbeiter*innen und der Führungskräfte. Die positiven Einflussfaktoren sind dabei mit einem Pluszeichen (+), negative Einflussfaktoren mit einem Minuszeichen (−) und Faktoren, die sowohl positiv als auch negativ wirken können, mit einem Plusminuszeichen (±) gekennzeichnet.

**Table 4:** 

	Mitarbeiter*innen	Führungskräfte
*Arbeitsmittel*	+ Technische Ausstattung im Homeoffice zum Teil besser als im Büro	+ Nötige Arbeitsmittel stehen zur Verfügung
	− Hohes individuelles Engagement, für gute technische Ausstattung zu sorgen	+ Neuwertige und ergonomische Ausstattung
	− Kein technischer Standard	− Zusätzlicher Zeit- und Stressfaktor durch Mitnahme von Technik aus Büro
	− Zusätzlicher Zeit- und Stressfaktor durch Mitnahme von Technik aus Büro	− Eigeninitiative für gute technische Ausstattung zu sorgen
	− Internetverbindung im Homeoffice manchmal problematisch	–
	− Fehlende Unterstützung bei Einrichtung des Heimarbeitsplatzes	–
*Arbeitsumfeld*	+ Umfeld im Homeoffice wird als sehr positiv empfunden	− Durch SARS-CoV-2-Arbeitsschutzverordnung leidet das Arbeitsumfeld
	− Mögliche Ablenkungen durch Kinder oder pflegebedürftige Personen im Homeoffice	+ Einsicht zur Notwendigkeit vorhanden
	− Zum Teil kein separates Arbeitszimmer zu Hause	–
*Arbeitsaufgabe*	± Neue Chancen und Herausforderungen durch starken Wandel der Arbeitsaufgabe	± Neue Chancen und Herausforderungen durch starken Wandel der Arbeitsaufgabe (Management zwischen Homeoffice und Präsenz; digitale Kommunikation)
	+ Schnellere Problemlösung per Telefon und E‑Mail	− Erhöhtes Stresserleben durch ungleiche Aufgabenverteilung und Zusatzaufgaben
	− Schwieriger Weg der Digitalisierung, Prozesse sollten vereinfacht werden	− Höheres Maß an Selbstorganisation erforderlich
	− Mögliche Ungleichbehandlung unter Kolleg*innen	− Skype als Zeitfresser und Störfaktor
*Arbeitsorganisation*	+ Bessere Arbeitsorganisation durch weniger Ablenkungen und Unterbrechungen	+ Gute Arbeitsorganisation durch eigene Kompetenzen möglich
	+ Verbessertes Gesundheitsempfinden durch gesteigerte Effektivität	–
	+ Energiegewinn durch Wegfall von Fahrtwegen und -zeiten	–
*Beziehung zu Kollegen*	− Starke Veränderung des Miteinanders	− Entfallen sozialer Aspekte und Teil des Teamgeistes
	− Eingeschränkter Kontakt zu Kollegen	− Konflikte durch fehlende Toleranz und Akzeptanz für Arbeit im Homeoffice
	− Soziale Aspekte entfallen	− Leistungsdruck und Wegfall von Ruhepausen durch Verlust der Zwischenmenschlichkeit
	− Neue Gangart muss gefunden werden	− Schwierige Einarbeitung neuer Kollegen über digitale Arbeitsmittel
	− Konflikte durch fehlende Toleranz und Akzeptanz für Arbeit im Homeoffice	–
	+ Trotzdem ein insgesamt gutes Teamgefüge	–
*Führung*	+ Gute Zusammenarbeit mit direkter Führung und Bereichsleitern	− Fehlende Unterstützung der Bereichs- und Teamleiter zu Beginn der Pandemie
	+ Guter Wechsel der Arbeitsweisen	− Fehlende Präsenz des Geschäftsführers und fehlendes Feedback
	− Fehlende Akzeptanz des Homeoffice	− Unzufriedenheit durch mangelnde Wertschätzung
	− Vorurteile bzgl. der Produktivität und Effizienz im Homeoffice	− Fehlende Stellungnahme der Geschäftsführung zum Homeoffice
	− Fehlende Präsenz des Geschäftsführers	− Fehlende Orientierung/fehlender Leitfaden zum Homeoffice
	−Fehlende Stellungnahme der Geschäftsführung zum Homeoffice	–

Im Auswertungsgespräch mit der Geschäftsführung und den Arbeitnehmervertretungen wurde aufgrund der Datenlage beschlossen, dass eine vertiefende Gesundheitswerkstatt in 3 Terminen angeboten wird, um mit Hilfe des Erfahrungswissens der verschiedenen Mitarbeiterebenen die bestehende Dienstvereinbarung zum mobilen Arbeiten zu aktualisieren. Hierbei konnten 31 Mitarbeiter*innen und 11 Führungskräfte erreicht und somit eine gemeinsame Unternehmensperspektive abgebildet werden.

## Diskussion

Grundsätzlich weichen die erhobenen Ergebnisse nicht von der anderer Autor*innen ab, die bereits vor der SARS-CoV-2-Pandemie Daten zum Arbeiten im Homeoffice erhoben worden sind [1, 12]. Das Arbeiten im Homeoffice stellt Anforderungen an die Beschäftigten (wie z. B. Eigenverantwortung, vermehrte Einzelarbeit und digitale Kommunikation), die wiederum positive als auch negative Beanspruchungsfolgen mit sich bringen [12]. So wurde z. B. in dieser Analyse das Gefühl von Autonomie ebenfalls als positiv beschrieben und als negative Beanspruchungsfolge der Technostress genannt. Interessant ist, dass im Vergleich zu aktuellen Studien die Mitarbeiter*innen dieser Befragung deutlich weniger negative Beanspruchungsfolgen in Bezug zu ihrer mentalen Gesundheit geschildert haben [11, 17]. Dies kann jedoch an der Stichprobengröße der Befragung liegen (siehe Limitationen der Arbeit).

Grundsätzlich sollte es das Ziel der Unternehmen sein, auf das bereits dargestellte Wissen zurückzugreifen und in einem ganzheitlichen Ansatz einen Rahmen für eine gesundheitsförderliche Gestaltung des mobilen Arbeitens zu schaffen [4].

Die SARS-CoV-2-Pandemie sollte hierbei als eine Chance genutzt werden, um Themen wie mobile Arbeit und das Arbeiten im Homeoffice weiter voranzubringen. Der aktuelle Forschungsbericht des Bundesministeriums für Arbeit und Soziales [5] zeigt, dass die Zahlen zum Arbeiten im Homeoffice leicht rückläufig sind. So befanden sich 42 % der Befragten im Zeitraum Juni bis Juli 2021 in der Homeoffice-Arbeit. Eine maximale Quote wurde Februar 2021 mit 49 % erreicht. Es hat fast die Hälfte der Befragten Erfahrungen zu dieser Arbeitsform gemacht, die sich auch langfristig auf ihre Einstellung zu ihrer Arbeit auswirken wird. Dies hatte sich auch in der vorliegenden Befragung gezeigt. Für die Teilnehmer*innen der Arbeitssituationsanalysen bestand der Wunsch, dass sie die Arbeit im Homeoffice in ihre Arbeitspraxis mit einfließen lassen möchten. Aus diesem Grund wird auch zum derzeitigen Zeitpunkt eine Aktualisierung der Dienstvereinbarung zum mobilen Arbeiten angestrebt.

Die ASITA kann hierbei für Unternehmen ein hilfreiches Tool sein, in einem überschaubaren zeitlichen Rahmen eine konkrete Vorstellung von der Arbeitssituation ihrer Mitarbeiter*innen zu bekommen. Im Vergleich zu einer klassischen Befragung können durch den Einsatz qualitativer Methoden, wie der ASITA, Ungereimtheiten geklärt, offene Fragen beantwortet und neue Perspektiven hervorgebracht werden. Erst mit einem qualitativen Verfahren ist es möglich, komplexe Wirkungsgefüge mit sämtlichen wirksamen, unabhängigen Variablen zu erfassen, um ein besseres Verständnis von der Arbeitssituation der Mitarbeiter*innen und Führungskräfte zu bekommen [13]. Der Einsatz der ASITA ist sehr vielfältig, und es können neben unterschiedlichen Fragestellungen auch unterschiedliche Branchen und Mitarbeitergruppen in Betracht gezogen werden [15]. Die Form der digitalen ASITA bietet sogar die Möglichkeit, Beschäftigte unabhängig von ihrem Beschäftigungsstandort (wie dem Homeoffice) partizipieren zu lassen. Zukünftig sollte die digitale ASITA auch für weitere Berufsgruppen der mobil-flexiblen Arbeit, z. B. für Beschäftigte im Außendienst, in Betracht gezogen werden. Hürden für die Umsetzung des Analyseinstruments könnten jedoch die technischen Voraussetzungen sein. Diese bilden eine essenzielle Basis, um den Ablauf der digitalen ASITA zu gewährleisten. Ebenfalls sollte bei der Umsetzung bedacht werden, dass soziale Aspekte, die sonst bei einem Workshop vor Ort nicht auffallen, wegfallen können. So kann es schwieriger sein, eine Vertrauensbasis bei den Mitarbeiter*innen für die Befragung aufzubauen. Des Weiteren ist es schwieriger, alle Beteiligten gleichermaßen in den qualitativen Teil der ASITA zu integrieren. Beteiligte, die sich nicht mit Redebeiträgen mitwirken wollen, gehen leichter in einer digitalen Befragung *verloren*. Ein Nachfragen oder aktives Ansprechen fällt in der digitalen Befragung deutlich schwerer, und somit können wichtige qualitative Daten und Perspektiven unerwähnt bleiben. Ebenfalls stellt die Wahrnehmung der Gruppendynamiken eine Herausforderung dar.

## Limitation der Arbeit

Bei einer Stichprobengröße von 16 Beschäftigten und der Wahl der Methode können auch stark individuell geprägte Ansichten einzelner Teilnehmer*innen das Gesamtbild der Auswertung beeinflussen. Um diesen Effekt entsprechend besser abfangen zu können, wurden die anschließenden Gesundheitswerkstätten mit einer größeren Teilnehmerzahl und vor allem auch mit Mitarbeiter*innen ohne Homeoffice-Erfahrungen durchgeführt. Für die Verwaltungsbehörde bildete dieses Vorgehen eine gute Entscheidungsgrundlage für die Weiterentwicklung der Dienstvereinbarung und ist somit eine gute Hilfestellung für die Praxis.

Im wissenschaftlichen Kontext kommt die vorgestellte Analysemethode an ihre Grenzen. So ist fraglich, ob sich auf dieser Basis Handlungsempfehlungen auf nationaler oder sogar auf internationaler Ebene ableiten lassen. Insbesondere die pandemische Situation hat sich in den einzelnen Ländern durch unterschiedliche Ausprägungen des Infektionsgeschehens auf Arbeitnehmer*innen teils unterschiedlich ausgewirkt, was sich auch in den Studienergebnissen zeigt [11, 17, 21].

## Fazit

Die digitale ASITA baut auf dem bekannten Wissen zur Erhebung der Arbeitssituation von Beschäftigten auf und bietet die Möglichkeit, Mitarbeiter*innen ortsunabhängig in dem ASITA-Prozess partizipieren zu lassen. Für das vorgestellte AOK-Gesundheitsprojekt war sie das Mittel der Wahl, um zeitnah die Arbeitssituation der Mitarbeiter*innen zu erfragen und gemeinsam nach einer zukünftigen Lösungsstrategie zu suchen.

Bei zukünftigen Umsetzungen dieses Formats sollten für jedes Unternehmen das Für und Wider der Methode betrachtet werden, damit diese zielgerecht eingesetzt werden kann. Es zeigt sich, dass sich das Analyseverfahren gut für die Praxis eignet, jedoch im wissenschaftlichen Bereich an seine Grenzen stößt.
